# Poor Eating Habits and Low Physical Activity Contribute to Weight Excess and Increase Cardiometabolic Risk in Adolescents Practicing Soccer as a Recreational Sport

**DOI:** 10.3390/children11070857

**Published:** 2024-07-15

**Authors:** Ikram Bezrati, Raouf Hammami, Halil İbrahim Ceylan, Karuppasamy Govindasamy, Mohamed K. Ben Fradj, Moncef Feki, Abderraouf Ben Mansour, Koulla Parpa

**Affiliations:** 1 Laboratory of Biochemistry, Faculty of Medicine of Tunis, Rabta Hospital, University of Tunis El Manar, LR99ES11, Tunis 1007, Tunisia; cnms@email.ati.tn (I.B.); utm@utm.tn (M.K.B.F.); moncef.feki@fmt.utm.tn (M.F.); bderraouf.benmansour@fmt.utm.tn (A.B.M.); 2 Tunisian Research Laboratory ‘Sports Performance Optimization’, National Center of Medicine and Science in Sports (CNMSS-LR09SEP01), Tunis 1003, Tunisia; crd@issepks.rnu.tn; 3 Higher Institute of Sport and Physical Education of Ksar-Said, Manouba University, Tunis 2010, Tunisia; 4 Department of Physical Education of Sports Teaching, Faculty of Sports Sciences, Atatürk University, Erzurum 25100, Türkiye; 5 Department of Physical Education and Sports Sciences, Faculty of Science and Humanities, SRM Institute of Science and Technology, Kattankulathur 603203, Tamil Nadu, India; gowthamadnivog@gmail.com; 6 Faculty of Sport and Exercise Science, UCLan University of Cyprus, Pyla 7080, Cyprus; kparpa@uclan.ac.uk

**Keywords:** cardiometabolic risk, childhood obesity, eating behavior, physical activity, overweight

## Abstract

Background: Monitoring anthropometry, dietary intake, and physical activity (PA) is essential to prevent/counteract childhood obesity. This study examined dietary intake, PA, and anthropometric characteristics in young boys practicing soccer as a recreational sport. Methods: A cross-sectional study included 226 boys aged 8 to 13 years participating in soccer as a recreational activity in football academies located in Tunis, Tunisia. Anthropometric measures allowed the calculation of body mass index, fat mass, and peak height velocity as markers of biological maturity. A three-day food record and a food frequency questionnaire estimated nutrient intake and eating habits. The International Physical Activity Questionnaire (IPAQ) was used to estimate the PA level of the participants. Results: It was found that a high percentage of the children had excess weight (54%) and excess fat mass (47%). The total energy, carbohydrate, and saturated fat intake of the children exceeded the recommended levels by approximately 10%, 15%, and 30%, respectively. However, the intake of unsaturated fat was below the estimated requirements, particularly in obese children. In addition to the unbalanced macronutrient intake, the children also showed an insufficient intake of many essential micronutrients. Around 60% to 70% of the children in all three groups had a low intake of magnesium, n-3 polyunsaturated fatty acids (PUFA), and vitamins B9, B12, and D. Moreover, 20% to 35% of the children in the three groups had an insufficient intake of vitamins A and C. Insufficient vitamin E intake was found in 63% of obese children and 35% of non-obese children. It was observed that the PA level was lower in the overweight/obese group compared to the normal-weight group (*p* < 0.005). More than three-quarters of overweight/obese children had low PA levels, about 20% were moderately active, and only 1 to 2% were highly active. Conversely, normal-weight children showed moderate to high PA levels. Conclusions: Poor eating behavior, an unbalanced diet, and a low PA level are prevalent in Tunisian boys practicing recreational sports. Such a combination is responsible for a disparity between energy intake and expenditure, contributing to weight excess and increased cardiometabolic risk. The study findings provide meaningful information for practitioners and authorities on applying a balanced diet and adequate PA to prevent and fight against obesity and improve cardiometabolic health in youth.

## 1. Introduction

Adequate dietary intake and physical activity (PA) are fundamental for maintaining optimal growth and health in children [1,2]. In transitional countries, where socioeconomic conditions, lifestyle, and eating habits are rapidly evolving, childhood obesity has become a prominent public health concern [3,4,5]. Tunisia exemplifies this trend, with recent studies indicating that overweight and obesity rates among children vary significantly depending on age, sex, and region, ranging between 11.6% to 48.9% and 2.7% to 10.0%, respectively [3,6]. This increasing prevalence is primarily attributed to heightened sedentary behaviors and reduced physical activity, leading to an imbalance between energy intake and expenditure [3,6,7].

Regular physical activity combined with a balanced diet plays a crucial role in preventing and managing excessive body weight by favorably impacting adiposity and physical fitness [8]. Despite these recommendations, a significant number of children fail to meet these activity levels, contributing to the global rise in childhood obesity.

In transitional countries like Tunisia, childhood obesity has become a prominent public health concern due to rapidly evolving socioeconomic conditions, lifestyle, and eating habits. The traditional consumption of fruits and vegetables is increasingly being supplanted by industrial foods, salty snacks, soft drinks, and sweets [7,9]. This shift is accompanied by a decline in the consumption of healthy, traditionally prepared dishes. Concurrently, children’s engagement in sedentary activities, such as watching TV and playing with electronic devices, has increased, leading to a reduction in physical exercise [10,11]. These behaviors not only contribute to obesity but also negatively impact cardiometabolic health [12].

While numerous studies have explored the issues of obesity and dietary intake among sedentary children and adolescents or those with low physical activity levels [13,14], there is a paucity of research focusing on diet patterns and physical activities among youth in underdeveloped and developing countries, including Tunisia. The existing literature often overlooks the dietary and physical activity behaviors of children engaged in recreational sports, a group that may exhibit different patterns of energy intake and expenditure.

The present study aims to fill this gap by investigating dietary intake and physical behaviors among Tunisian youth who practice soccer as a recreational sport. Despite their engagement in physical activities, these children may still experience unbalanced energy intake and expenditure, increasing their risk of overweight, obesity, and cardiometabolic health alteration. We hypothesize that despite their engagement in physical activities, these children may still experience unbalanced energy intake and expenditure, increasing their risk of overweight, obesity, and cardiometabolic health alterations. This research is critical for developing targeted interventions to promote healthy lifestyle choices and prevent obesity in this population. Understanding the interplay between dietary intake and physical activity in children is essential for addressing the growing issue of childhood obesity, especially in transitional countries. While examining the specific behaviors and patterns of Tunisian youth engaged in recreational sports, this study aims to provide insights that can inform public health strategies and promote better health outcomes for children in similar contexts.

## 2. Materials and Methods

### 2.1. Study Design and Participants

A cross-sectional study involved boys attending two private football academies in Tunis (Tunisia). To be included in the study, participants must be male and aged between 8 and 13 years. They should have actively participated in recreational soccer for at least one year and attended at least 80% of training sessions and matches. In addition, participants must be in good general health with no chronic illnesses or injuries that could affect their ability to engage in physical activity. They should follow a regular training schedule, practicing soccer at least twice a week. Lastly, participants must not have used any performance-enhancing substances or medications that could affect their physical activity or metabolic measures. Of the 243 eligible children, eight boys did not meet the inclusion criteria, and nine were excluded. A total of 226 participants were retained for analysis. Written informed consent was obtained from participants’ parents or guardians. The study was conducted according to the requirements of the Declaration of Helsinki and was approved by the Laboratory of Biochemistry, Ethics Committee of the Rabta Hospital (Code: LR99ES11, 2 April 2021). To ensure confidentiality, all data were anonymized. Participants underwent two 45 min football training sessions in the academy and two 45 min sessions of school-based physical education, weekly. No specific dietary recommendations were delivered at the inclusion.

### 2.2. Procedures

The procedures were conducted indoors, consistently scheduled between 08.00 a.m. and 10.00 a.m. each day, and under controlled environmental conditions (temperature: 18–21 °C, humidity: 45–55%). All procedures took place during the second half of the competitive season (March–May 2022). To ensure participants were well prepared, a familiarization session was held one week before the commencement of the study, during which youth players were acquainted with the tests and questionnaires to be administered. Participants received detailed instructions regarding the procedures. The order of tests remained consistent across participants, beginning with a medical examination, followed by anthropometric measurements, blood sampling, a physical activity questionnaire, and concluding with a nutritional inquiry.

### 2.3. Anthropometric Measures

A trained sports science professional measured body weight and height following the guidelines set forth by the International Society for the Advancement of Kinanthropometry (ISAK) [15]. Body mass was assessed with a precision electronic digital scale, ensuring participants were barefoot and minimally clothed. Measurements were recorded to the nearest 0.01 kg, following previously established standardized protocols [16], and height was measured using a calibrated stadiometer, allowing the calculation of the body mass index (BMI). Participants were categorized into three groups based on the WHO child growth standards for BMI [17]: the normal-weight group (NWG, BMI ≤ 85th percentile), the overweight group (OWG, 85th percentile < BMI < 97th percentile), and the obese group (OBG, BMI > 97th percentile). Triceps and subscapular skinfold thickness were measured using Harpenden skinfold calipers (Baty International, West Sussex, England). Body fat percentage was estimated using Slaughter’s prediction equation [18]. Body fat excess/adiposity was defined as body fat percentage > 75th percentile in American children [19]. The Mirwald equation for boys [20] estimates years from peak height velocity (PHV) as an indicator for the adolescent growth spurt.

### 2.4. Dietary Assessment

Dietary intake was evaluated using a 3-day food record (covering two weekdays and one weekend day) and a food frequency questionnaire (FFQ). The nutritional inquiry was administered by an experimented nutritionist (IB) in the presence of one parent or guardian. For accuracy in estimating food portions, pictures of food and typical Tunisian food preparations were used. The total energy intake amounts of macronutrients (carbohydrates, fat, proteins) and micronutrients (fatty acids, minerals, and vitamins) were calculated using nutrition analyzing software (Nutri Pro7, CERDEN, Brussels, Belgium version 2011). Nutrient intake was classified as low, adequate, or high based on American references [21]. Macronutrient intakes were compared to the “acceptable macronutrient distribution range”. For micronutrients, the “estimated average requirement” was considered as the lower limit, and the “recommended dietary allowances (RDA)” were the upper limit of normal intake. For total energy, the “estimated energy requirement” was considered the lower limit, and the RDA was the upper limit of normal intake. For each nutrient, values below the low threshold defined low intake, and values above the top threshold defined high intake, whereas intake within the interval was considered adequate. A validated FFQ was used to establish food typology [22]. The FFQ was translated from English into Arabic and was approved by a panel of Tunisian nutritional experts. It consisted of 15 items enquiring about the frequency of consumption of food groups including milk/dairy products, meat, fish, egg, fruits, vegetables, bread and pasta, soft drinks, sweets, chocolates, biscuits, pastries, fries, and fast foods (pizza, hamburgers).

### 2.5. Physical Activity and Energy Need Measurement

The levels of physical activity (PA) of participants were identified using the International Physical Activity Questionnaire (IPAQ) adapted for adolescents [23]. The questionnaire was administered by one investigator (IB) to the children in the presence of one parent. The IPAQ inquired about the average duration of five categories of PA over 24 hours during the previous week. For each category, the duration in hours is multiplied by a specific coefficient. The coefficient is as follows: 1 for activities performed in the supine position, 1.5 for activities performed in a seated position, 2.2 for activities performed in a standing position, 3.5 for slight physical activities, and 5 for moderate-to-vigorous physical activities. The points are divided by 24 to obtain an average score per hour. PA is considered low for a score below 1.5, moderate for a score between 1.6 and 1.7, and high for a score above 1.8 [24]. Energy needs were estimated by multiplying the basal metabolic rate by the level of PA and adding requirements for growth. The basal metabolic rate was calculated using Black’s formula for healthy males under 75 years [25].

### 2.6. Biochemical Analyses

Blood samples were collected following a 10−12 h overnight fast. Post-collection, the samples were subjected to centrifugation at 2000× *g* for 20 min. The resulting plasma was then frozen at −40 °C and stored for up to three months before analysis. Plasma glucose, creatinine, uric acid, total and HDL cholesterol, triglycerides, calcium, phosphorus, iron, total proteins, the C-reactive protein (CRP), and insulin were assessed on an Architect Ci8200 analyzer (Abbott Laboratories, Abbott Park, IL, USA) using the respective reagents kits. Hemoglobin was analyzed by a colorimetric method on an XN-2000^MC^ Hematology analyzer (Sysmex, Kobe, Japan). The homeostasis model assessment of insulin resistance (HOMA-IR) was determined using the following formula: HOMA-IR = [(fasting insulin in µU/mL) − (fasting glucose in mg/dL)]/405] [26].

### 2.7. Statistical Analysis

The statistical analysis was performed utilizing SPSS for Windows software (version 18.0). Descriptive statistics are presented as the mean ± standard deviation (SD), median with interquartile range (IQR), or percentages, as applicable. The Shapiro−Wilk test was employed to evaluate the normality of continuous variables. Comparative analyses between groups were executed using Analysis of Variance (ANOVA) or the Mann−Whitney U test for continuous variables, and the chi-square test for categorical variables. A *p*-value of less than 0.05 was considered indicative of statistical significance.

## 3. Results

### 3.1. Main Characteristics of Participants

Among the 226 children included in the study, 121 children (53.5%) had body mass excess, with 50 overweight children (22.1%) and 71 obese children (31.4%). Body fat excess was seen in 106 children, representing 46.9% of participants (16.19% in the NWG, 73.51% in the OWG, and 93.06% in the OBG). According to maturity status, 81.5% of children were below PHV, 16.6% were around PHV, and 1.9% were above PHV. Plasma cholesterol, triglycerides, uric acid, CRP, insulin, and HOMA-IR were significantly higher in OWG and OBG compared to NWG (Table 1).

### 3.2. Energy and Dietary Intake in Participants

**Figure 1:**
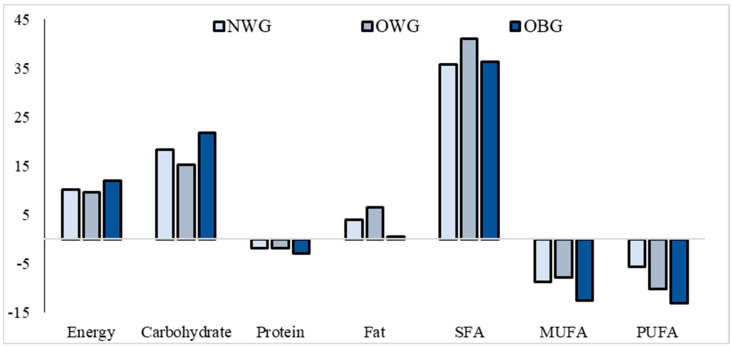
Percentage of difference in energy/macronutrients dietary intakes and estimated needs among children according to body composition. Notes: MUFA, monounsaturated fatty acids; NWG, normal-weight group; OBG, obese group; OWG, overweight group; PUFA, polyunsaturated fatty acids; SFA, saturated fatty acids.

**Figure 2:**
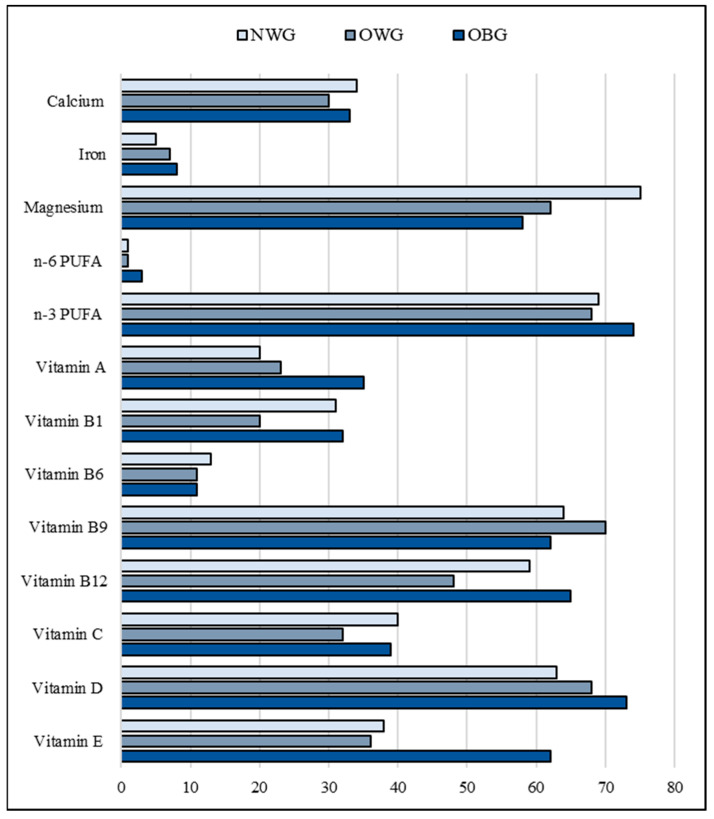
Selected micronutrient deficiency rates in children according to body composition. Notes: NWG, normal-weight group; OBG, obese group; OWG, overweight group; PUFA, polyunsaturated fatty acids.

Total energy, carbohydrates, and saturated fat intakes exceeded the requirements in the three groups of children by around 10%, 15%, and 30%, respectively. On the contrary, unsaturated fat intake was under the estimated needs, markedly in the obese children (Table 2, Figure 1). Besides an unbalanced macronutrient intake, the children showed an insufficient intake of many essential micronutrients. Specifically, the prevalence of low magnesium, n-3 polyunsaturated fatty acids (PUFA), and vitamins B9, B12, and D intake ranges between 60% and 70% in the three groups. An insufficient intake of vitamins A and C was found among 20 to 35% of the three groups of children. Vitamin E insufficient intake was found in 63% and 35% of obese and non-obese children, respectively (Figure 2).

**Figure 3:**
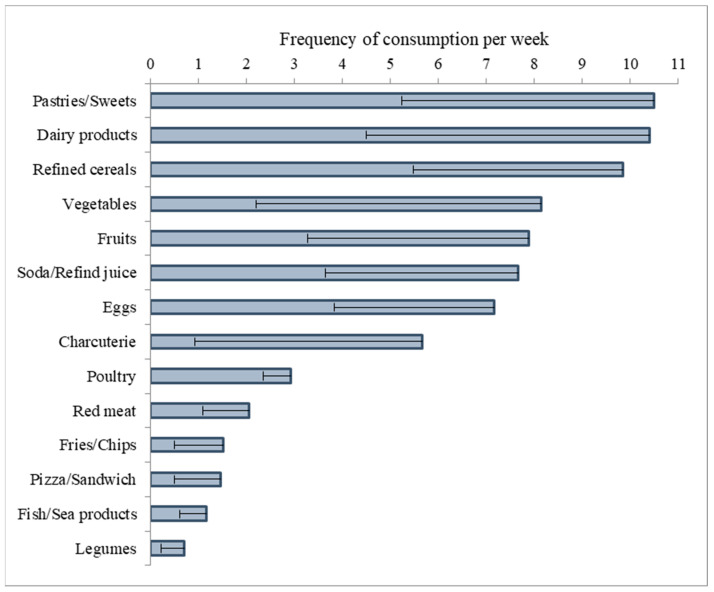
Frequency of consumption of food categories per week in overweight/obese children.

Almost all overweight/obese children consume milk/dairy products, refined cereals/pasta, pastries/sweets, and soft drinks at least once daily. Most children consume vegetables and fruits daily, but they often eat one or two servings of each group, daily. Most children eat eggs daily, meat or poultry more than twice, and fish once, weekly. Most overweight/obese children consume fries, chips, pizza, and fast foods at least once per week. Finally, the consumption of legumes is low in most of these children (Figure 3).

### 3.3. Physical Activity Level of Participants

**Figure 4:**
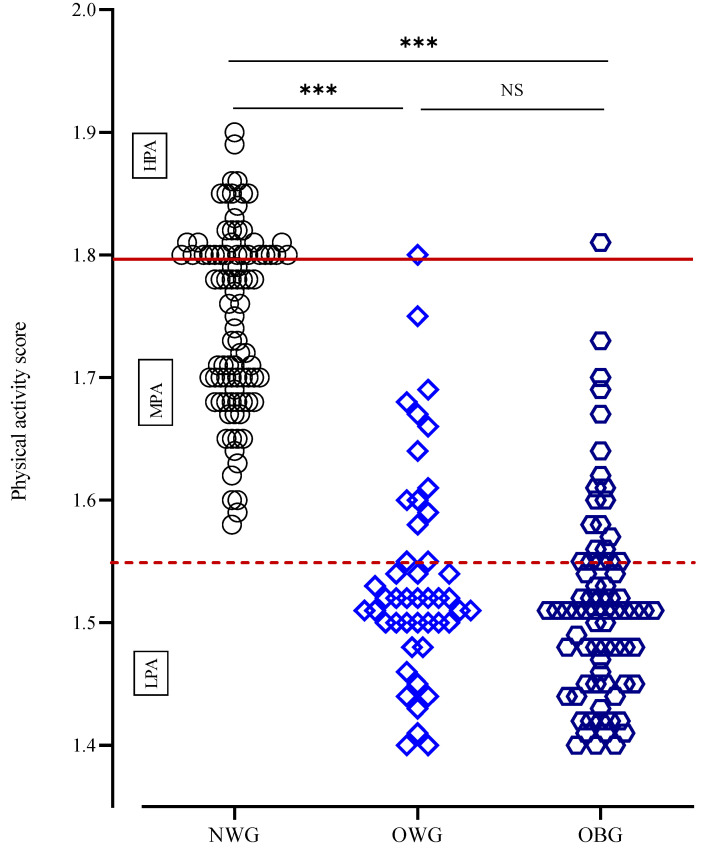
Distribution of PA score in children according to body composition. Notes: HPA, high PA; LPA, low PA; MPA, moderate PA; NWG, normal-weight group; OBG, obese group; OWG, overweight group. ***: *p* < 0.001, NS: *p* > 0.05.

PA level was significantly lower in OWG and OBG compared to NWG. Above three-quarters of overweight/obese children showed low PA levels, around 20% were moderately active, and only 1 to 2% were highly active. Normal-weight children showed moderate to high PA levels (Table 1, Figure 4).

## 4. Discussion

The study revealed a high prevalence of overweight and obesity among these ostensibly active children. Almost all children have poor eating habits and those with excess weight are insufficiently active and show increased cardiometabolic risk markers. The children of the three groups showed poor eating behavior with high consumption of high-density energy foods (i.e., carbohydrates and saturated) and low consumption of vitamin-rich foods. PA was low in almost all overweight/obese children, but moderate to high in normal-weight children. The study findings confirm the hypothesis that unbalanced energy intake and expenditure are responsible for excess weight and increased cardiometabolic risk.

The high rate of overweight/obesity in these children is a worrying trend. However, this is part of the global pandemic of obesity affecting both the developed and developing world [27,28,29]. Obesity is a complex and multi-factorial problem that comprises metabolic, behavioral, psychosocial, and genetic factors, and it can compromise the psycho-physical health of adolescents [30]. The speed with which the prevalence of obesity has increased suggests that it is rather related to incoming factors such as unhealthy eating and sedentary behavior. Excessive energy intake and reduced PA are key factors in the pathogenesis of obesity [31,32]. In these children, excess energy and carbohydrate intake are due to the frequent consumption of soft drinks and refined cereals. Carbohydrates are essential for optimal PA as they improve the ability to perform strenuous exercises [33] and delay the onset of muscle fatigue [34]. However, a high intake of high-glycemic load foods promotes lipogenesis and obesity. Their sweet taste enhances the palatability of food and induces a passive overconsumption of energy [35]. In these children, fat intake is in line with requirements but is unbalanced towards saturated fats at the expense of unsaturated fats. Due to high energy density, flavor, palatability, reduced oxidative capacity, and high storage capacity, saturated fats contribute to obesity [36]. PUFA intake was below the requirements in overweight/obese children. As precursors of active compounds such as prostaglandins, leukotrienes, lipoxins, and resolvins, PUFA modulates inflammation and influences health [37]; n-6 PUFA derivatives are pro-inflammatory mediators while n-3 derivatives have anti-inflammatory/pro-resolving potential [38,39]. Accordingly, a high n-6:n-3 ratio in the diet is considered unhealthy. In this study, PUFA intake was unbalanced in favor of n-6 derivatives, which may affect children’s health. Adopting an n-3 PUFA-rich diet containing more fish and other marine products could help improve children’s metabolic health. Protein intake was equivalent to requirements in normal-weight and overweight children but exceeded requirements in obese children. Sufficient protein intake is necessary for normal growth. However, high protein intake during childhood could induce early adipocyte proliferation, thus promoting adipogenesis [40]. In contrast to a plentiful intake of energy and macronutrients, children showed low intakes of micronutrients such as magnesium, calcium, and vitamins B9, B12, D, and E. This is explained by the low consumption of fruit, vegetables, legumes, and red meat, a common nutritional problem in children from developing countries [41]. These nutrients are involved in energy metabolism, the synthesis of proteins and nucleic acids, and maintaining normal immune and nervous functions. Their deficits might alter cellular metabolism, harm growth, and hinder neuro-musculoskeletal and immune functions [42,43]. Replacing energy-dense foods with fruit and vegetables may help to reduce weight, prevent micronutrient deficiencies, and improve health [44].

Our study showed that PA levels are low in overweight/obese children but adequate in normal-weight children. The children are engaged in sports and are supposed to be physically active. However, with four weekly physical exercise sessions, overweight/obese children do not reach adequate PA levels for several reasons. Due to their low physical power, reduced cardiorespiratory fitness, and poor sporting performance, these children are less interested in physical activity and sports. They spend their leisure time playing on electronic devices and watching TV rather than practicing outdoor activities. They are pushed by their parents to enroll in the academy, but repeatedly miss training sessions and rarely participate in competitions. During the sporting sessions, overweight/obese children tend to tire quickly, so they reduce or stop their efforts. Consequently, they move less, exert less physical work, and spend less energy than their normal-weight peers. As these underactive children have poor eating behavior and excessive energy intake like their physically active normal-weight peers, we postulate that insufficient PA is the main determinant of excess weight in them [32]. Low PA associated with poor eating habits is responsible for energy intake and expenditure imbalance that promotes adiposity and obesity [45].

There is accumulating evidence for a relationship between PA and metabolic health. High sedentary time and screen time are associated with an unhealthy cardiometabolic profile [45]. PA exhibits an inverse relationship with triglyceride and low-density lipoprotein (LDL) cholesterol levels while demonstrating a direct correlation with high-density lipoprotein (HDL) cholesterol levels. [46]. For instance, Viitasalo et al. [47] found that unstructured and low levels of PA are inversely correlated with fasting insulin levels. In contrast, sufficient PA improves insulin sensitivity by reducing body fat in children [48,49]. The current study revealed increased plasma total cholesterol, triglycerides, uric acid, the CRP, insulin, and HOMA-IR in underactive overweight/obese children, indicating a higher cardiometabolic risk in them. An adverse cardiometabolic profile during childhood, including a higher BMI and body fat percentage, along with lower aerobic fitness, has been linked to an increased risk of developing metabolic diseases such as obesity, type 2 diabetes, and cardiometabolic diseases in adulthood [50]. Consequently, fostering adequate PA and nutritious dietary habits from an early age is crucial to mitigating the risk of life-threatening diseases in adulthood.

The physical maturation of children and adolescents can potentially affect their body composition. In this study, the majority of children were not yet in puberty, and there were no differences in the maturation stage based on body mass. Therefore, it is unlikely that physical maturation influenced the study’s findings.

The present study has strengths and limitations. In our study, a significant number of participants were involved. We determined the nutritional needs of each participant by taking into account their body mass, physical activity level, and growth requirements. We defined body composition according to international guidelines. However, the levels of PA of participants were identified using the IPAQ adapted for adolescents. While the use of questionnaires has limitations in accurately capturing PA levels, this method was chosen due to its feasibility and cost-effectiveness in large-scale studies. Future research should consider employing objective measures, such as accelerometers, to provide more accurate assessments of PA. Moreover, fat mass was evaluated based on the skinfold measures method, which is less precise than DEXA and MRI. The software (Nutri Pro7, CERDEN, Brussels, Belgium version 2011) used to estimate nutritional intake is more suited for European than Tunisian foodstuff. Since some Tunisian traditional dishes were absent from the database, we introduced meal ingredients into the nutritional program, which could have resulted in a less accurate estimation. Finally, the study participants are issued from relatively underprivileged families, which prevents the generalization of the conclusions. Additionally, the study could not investigate the underlying mechanisms responsible for the imbalance between energy intake and expenditure, which heightens the risk of overweight, obesity, and cardiometabolic health issues in children. This limitation was due to the absence of hormonal and immunological testing apparatus and biomechanical analysis, which are necessary for a comprehensive understanding of the changes observed in physical fitness measures. However, the findings may apply to children from transitional countries with similar characteristics.

The findings of this study have significant practical implications for coaches, parents, and healthcare providers involved in youth sports. Understanding the dietary intake and physical activity behaviors of Tunisian youth engaged in recreational soccer can help in developing targeted interventions to promote healthier lifestyles and prevent obesity and related cardiometabolic risks. Coaches can use these insights to design training programs that incorporate nutritional education and monitoring, emphasizing balanced energy intake and expenditure to optimize performance and health outcomes. Parents can benefit from understanding the importance of balanced nutrition and regular physical activity for their children’s health and can be provided with educational resources to guide healthier food choices and encourage active lifestyles. Healthcare providers can use the study’s data to better assess and address the health needs of young athletes, incorporating regular monitoring of dietary habits and physical activity levels into routine check-ups for the early identification of potential health issues. Additionally, the study underscores the need for policymakers to support programs that promote physical activity and healthy eating among youth, encouraging schools and community organizations to implement comprehensive health programs that include education on nutrition and exercise. By addressing these practical aspects, the study contributes to a holistic approach to managing and preventing obesity and cardiometabolic risks in youth, ultimately leading to improved health outcomes and well-being. Future longitudinal studies that track changes in dietary intake, physical activity, and health outcomes over time would provide valuable insights into the long-term effects of participating in recreational sports on youth health. Additionally, exploring the impact of specific dietary interventions and structured physical activity programs tailored to the needs of young athletes could help in developing effective strategies for preventing obesity and promoting health. It would also be beneficial to investigate the role of psychological factors, such as motivation, emotions, and self-efficacy, in influencing dietary and physical activity behaviors. Lastly, examining the socioeconomic and cultural factors that influence health behaviors in different populations could offer a more comprehensive understanding of how to support healthy development in youth.

## 5. Conclusions

The study showed that overweight and obesity are common in supposedly active children. Dietary patterns are characterized by a high intake of high-energy-density foods and a low intake of vitamins- and minerals-rich foods. PA is insufficient in overweight/obese children and is likely the main determinant of weight excess and increased cardiometabolic risk in children. A strategy aiming at promoting PA and healthy eating at an early age is required to improve cardiometabolic profiles and prevent life-threatening diseases in adulthood. Such a strategy consists of replacing dense food rich in sugars and saturated fat with fish, vegetables, and fruit (i.e., a return to traditional food patterns) and ensuring that children, particularly those with weight excess, engage in regular and sufficient PA.

## Figures and Tables

**Table 1:** Anthropometrical and biochemical characteristics and physical activity level in children according to body mass class (*n* = 226).

	NWG (*n* = 105)	OWG (*n* = 49)	OBG (*n* = 72)	*p*
Age, years	11.0 ± 2.02	10.7 ± 2.03	11.5 ± 2.01	0.11
Body mass, kg	32.8 ± 7.75	42.8 ± 10.1	56.5 ± 15.6	<0.001
Fat mass, %	17.8 ± 5.08	26.1 ± 5.24	43.6 ± 5.81	<0.001
Fat mass excess, %	16.19	75.51	93.06	<0.001
Maturity status				0.55
Below PHV	81.5	82.8	83.6	
Around PHV	16.6	15.2	15.0	
Above PHV	1.9	2.0	1.4	
PA level score	1.76 ± 0.068	1.51 ± 0.056	1.49 ± 0.058	<0.001
PA level class, %				<0.001
Low	0	76.0	77.8	
Moderate	52.6	22.4	20.8	
High	47.4	2.0	1.4	
Blood sugar, mg/dL	89.9 ± 6.8	89.6 ± 7.8	91.3 ± 7.0	0.34
Creatinine, mg/L	6.16 (0.77)	6.31 (0.81)	6.27 (0.76)	0.29
Uric acid, mg/L	36.5 ± 8.2	38.7 ± 9.9	42.5 ± 12.3	<0.001
Total cholesterol, mg/dL	153 ± 23.2	154 ± 24.4	164 ± 31.7	0.02
HDL cholesterol, mg/dL	54.0 ± 10.2	52.0 ± 10.2	52.0 ± 11.7	0.365
Triglycerides, mg/dL	55.6 ± 18.9	69.9 ± 32.5	71.7 ± 26.4	<0.001
Ca, mg/L	95.1 ± 4.12	95.8 ± 3.76	96.2 ± 3.87	0.15
P, mg/L	42.5 ± 3.97	42.8 ± 5.0	43.6 ± 4.48	0.7
Mg, mg/L	21.7 ± 2.51	21.3 ± 1.89	22.1 ± 1.88	0.76
Iron, mg/L	73.2 ± 4.71	73.7 ± 3.69	73.5 ± 3.95	0.76
Total proteins, g/L	73.2 ± 4.71	73.7 ± 3.67	73.5 ± 3.94	0.77
CRP, mg/L	0.45 (0.67)	0.61 (2.71)	0.95 (2.32)	<0.001
Insulin, UI/L	5.42 (3.66)	8.54 (6.04)	11.7 (9.17)	<0.001
IR	1.12 (0.83)	1.35 (1.46)	2.67 (2.17)	<0.001
Hemoglobin, g/L	126 ± 11.2	130 ± 10.1	130 ± 10.9	0.12

Notes: IR: insulin resistance; Ca: calcium; CRP: C-reactive protein; P: phosphorus; Mg: magnesium; NWG, normal-weight group; PA, physical activity; PHV, peak height velocity; OBG, obese group; OWG, overweight group.

**Table 2:** Daily energy and nutrient intake compared to correspondent estimated needs in participants by BMI (*n* = 226).

	Normal-Weighed Group (*n* = 105)		Overweighed Group (*n* = 49)		Obese Group (*n* = 72)	
	Estimated Needs	Dietary Intake	Estimated Needs	Dietary Intake	Estimated Needs	Dietary Intake
Energy, kcal/d	2297 ± 281	2529 ± 600 **	2440 ± 277	2672 ± 741 **	2538 ± 369	2843 ± 809 **
Proteins, g/d	104 ± 12.7	102 ± 17.7	110 ± 12.5	108 ± 19.6	114 ± 15	110 ± 19.1
Carbohydrate, g/d	267 ± 37.6	316 ± 82.4 ***	289 ± 40.0	333 ± 115**	299 ± 45.5	364 ± 120 ***
Fat, g/d	90.5 ± 12.9	94.1 ± 29.4	93.9 ± 12.5	100 ± 31.5	98.2 ± 13.8	98.7 ± 37.2
SFA, g/d	26.8 ± 3.3	36.4 ± 13.2 ***	28.1 ± 3.4	39.6 ± 18.4 **	29.2 ± 4.2	39.8 ± 17.0 ***
MUFA, g/d	40.1 ± 5.1	36.6 ± 13.1 *	42.4 ± 5.1	39.1 ± 12.2	43.9 ± 6.1	38.4 ± 14.3 **
PUFA, g/d	22.5 ± 3.1	21.2 ± 9.1	23.6 ± 2.9	21.2 ± 6.5	24.2 ± 3.3	21.0 ± 8.2 **
n-6 PUFA, g/d	18.1 ± 2.1	18.4 ± 7.0	18.1 ± 2.2	17.9 ± 6.1	19.6 ± 2.7	17.6 ± 7.4
n-3 PUFA, g/d	4.4 ± 0.6	3.1 ± 1.5 ***	4.7 ± 0.6	3.3 ± 1.8 ***	4.8 ± 0.7	3.3 ± 1.8 ***

Notes: SFA, saturated fatty acids; MUFA, monounsaturated fatty acids; PUFA, polyunsaturated fatty acids; *, *p* < 0.05; **, *p* < 0.01; ***, *p* < 0.001 (compared to estimated needs in the correspondent body mass class).

## Data Availability

Data can be obtained by contacting the lead author.
